# Prosociality during COVID‐19: Globally focussed solidarity brings greater benefits than nationally focussed solidarity

**DOI:** 10.1002/casp.2553

**Published:** 2021-06-16

**Authors:** Hanna Zagefka

**Affiliations:** ^1^ Department of Psychology Royal Holloway, University of London Egham UK

**Keywords:** common fate, COVID‐19, donation, identification with all of humanity, intergroup helping, prosociality, solidarity

## Abstract

Many charities are appealing for donations to address problems caused by the COVID‐19 crisis. Two survey studies (total *N* = 500) tested predictors of donation intentions of British participants to help those suffering from the crisis in Britain (ingroup donations) and overseas (outgroup donations). Perceptions of international, global common fate in our success in managing and overcoming the crisis was positively associated with prosocial intentions towards both national ingroup and outgroup targets. In contrast, a desire to ‘close ranks’ within the national ingroup in the face of the pandemic threat was associated with more prosocial intentions towards national ingroup targets only, but it was associated with *fewer* prosocial intentions towards outgroup targets. This suggests that a focus on global solidarity (in the form of global common fate and identification with all humans) has positive effects on helping both within and across group boundaries, whereas a focus on national solidarity (in the form of ‘closing ranks’ in the face of the pandemic threat) has positive effects on helping within the national group but negative effects on prosocial tendencies towards outgroup members. The effect of perceived global common fate on both ingroup and outgroup helping was mediated by identification with all of humanity. Findings are discussed in terms of practical implications for managing the COVID‐19 crisis. Please refer to the Supplementary Material section to find this article's Community and Social Impact Statement.

## 1 INTRODUCTION

The COVID‐19 pandemic has been gripping the globe for an extended period of time, and this is posing unprecedented challenges for the charity sector. Many people are feeling the impact of the lockdown‐induced economic downturn on their personal finances. Charitable donations have declined (Charities Aid Foundation, 2020), making competition for funding tougher. At the same time, the need of those suffering from the economic and health impacts of the pandemic, particularly among those who were already disadvantaged before the crisis, is growing. The goal of this work was to investigate what motivates people to donate money to help others suffering from the impacts of the COVID‐19 pandemic—both those who are members of the national ingroup and those who are members of national outgroups. There is a wealth of fundraising appeals addressing coronavirus‐related problems both domestically and internationally, and this work set out to test predictors of donating to causes at home and abroad. Considered were the effects of a perception of global common fate in managing the pandemic, potential effects of this on identification with all of humanity, and effects of a desire to ‘close ranks’ at the national level to manage the crisis. As such, a focus on global solidarity was juxtapositioned with a focus on national solidarity, to chart their effects on prosocial behaviours within and across national group boundaries.

### 1.1 Theoretical background: Group identities and helping

The theoretical foundations to this investigation lie in the literature on social identities, group processes, and prosocial behaviour. There are three ways that are relevant here in which social identities inform prosocial behaviour. First, in categorical terms, people are more likely to help ingroup members than outgroup members. By and large, help is more readily forthcoming for others that are part of the social ingroup (Bloom, 2017; Stuermer & Snyder, 2010; van Leeuwen & Zagefka, 2017). For example, those who incorporate other humans across ethnic, religious, and national boundaries into their ‘moral ingroup’ are more likely to offer help, even in the face of potentially great personal sacrifice, as has been demonstrated in studies of those who assisted Jewish people in Nazi‐occupied Europe (Reykowski, 2002). Ingroup bias in donation decisions has also been demonstrated in the context of COVID‐19 (Li, Li, Jiang, & Liu, 2020). Of course, people do, on occasion, also extend kindness towards outgroup members (Sierksma, Thijs, & Verkuyten, 2015; Stürmer, Snyder, & Omoto, 2005; Vezzali, Cadamuro, Versari, Giovannini, & Trifiletti, 2015), but generally ingroup helping is more easily triggered. Thus, whether someone is seen as an ingroup member or an outgroup member affects the likelihood that this person will be helped when in need.

Second, in correlational terms, the more people identify with and are psychologically engaged in their ingroup, the more important that group is to their sense of self, and the more they are motivated to help others in that group. Strength of identification with social categories clearly affects helping decisions, in the form of donations and other prosocial behaviours (e.g., Chapman, Masser, & Louis, 2020; Drury et al., 2019). Thus, helping decisions are not only affected by categorical ‘ingroup versus outgroup’ categorization of a given target. Rather, once a target has been categorized as ‘ingroup’, the *extent* of the potential helper's identification with the common identity also matters. There is clear evidence that greater perceived oneness between self and other (Cialdini, Brown, Lewis, & Neuberg, 1997) and greater fusion with the group (Swann, Gómez, Huici, Morales, & Hixon, 2010) increase ingroup helping. Ingroup identification has been shown to be linked to behaviours designed to protect other ingroup members also in the context of COVID‐19 (Liekefett & Becker, 2021). Thus, the more strongly someone is identified with their group, the more likely is it that this person will feel moved to act prosocially towards other ingroup members.

Third, group memberships are malleable and depend on situational salience and context (Jetten, Reicher, Haslam, & Cruwys, 2020; Turner, 1987). New shared identities can be created and arise from novel circumstances, such as shared adversity (Ntontis, Drury, Amlôt, Rubin, & Williams, 2020; see also Solnit, 2010). Thus, if identities are pliable, and if they are related to prosociality, this opens the opportunity to use them as a lever to increase prosociality and solidarity in the face of humanitarian emergencies such as the COVID‐19 pandemic.

To be sure, there are many factors other than those related to identity that affect whether prosocial behaviour will be displayed. For example, there might be individual differences in values and prosocial tendencies (Politi, Van Assche, Caprara, & Phalet, 2021) that impact on decisions to help. In this current article, we chose to focus on variables related to identities and global and national solidarity for two reasons. First, at least in Britain, appeals to national solidarity were featured frequently in the media and in soundbites by politicians at the start of the pandemic and throughout, and we were interested in studying the effects of this. For example, citizens were told to comply with social distancing rules for the sake of ‘Queen and country’ (The Economist, 2020), to ‘protect the NHS’ (with the NHS being the National Health Service, a cherished national institution subject of much national pride, Department of Health and Social Care, 2021), and to display ‘national unity’ (BBC, 2020b).

Second, there was growing concern among health professionals about a lack of global solidarity, for example, in the form of ‘vaccine nationalism’ (e.g., The Guardian, 2021), and we were interested in the psychological processes that might help address this problem. For example, the WHO warned that the world's poorest risk being ‘trampled in stampede for vaccines’ (The Telegraph, 2020). Hence, there seemed to be a mismatch between the nationally focussed rhetoric employed by politicians, and the need for a global and international perspective highlighted as necessary by health experts (e.g., The Guardian, 2020).

For these reasons, the focus of this work was on variables related to global solidarity, in the form of perceived common fate and identification with all humanity, and national solidarity, in the form of a desire to ‘close ranks’ with other national ingroup members in the light of the pandemic threat. In this way, the effects of global solidarity was juxtapositioned with the effects of national solidarity.

### 1.2 Factors related to global solidarity: Perceived global common fate, and identification with all humanity

There are reasons to propose that perceived global common fate and shared threat will increase identification with others who share the same fate, and through this with help extended to those others, also in the context of the COVID‐19 crisis. As demonstrated in the context of many disasters, suffering from the same adversity can create new shared identities and solidarity with others who would formerly have been seen as outgroup members (Bowe et al., 2021; Ntontis et al. 2021; Solnit, 2010). For example, in the context of a flooded community, common fate was positively associated with an emerging sense of community and identification with the group, as well as with social support (Ntontis et al., 2020).

Support can also be gleamed from the results of some classic experiments: Schachter (1959) demonstrated that participants induced to be jointly anxious were more likely to seek out the company of others, confirming the effect of shared threat on affiliation‐seeking behaviour. A number of other theoretical approaches have emphasized joint threat, uncertainty and anxiety as important motives for seeking group belonging and affiliation (e.g., Baumeister & Leary, 1995). The fact that perceived common fate can give rise to a sense of shared identity is supported by evidence that suggests that a common threat invokes recategorization, so that those formerly seen as outgroup members become part of the common ingroup (Flade, Klar, & Imhoff, 2019), and it is supported by evidence that shows that a common perceived threat increases identification with others exposed to this threat (Schmid & Muldoon, 2015). The fact that perceived common fate is linked to not only to affiliation‐seeking but also to helping for fellow sufferers is further supported by laboratory studies which find that participants are more likely to help others who are facing a similar fate to themselves (Dovidio & Morris, 1975). Other work, too, supports the notion that shared threat motivates ingroup strengthening behaviours (Wohl, Branscombe, & Reysen, 2010).

The findings reviewed above speak to an effect of ‘common fate’ on ‘identification’ and to an effect of ‘common fate’ on ‘helping’. In addition, as also reviewed above there is evidence that shared group membership and ‘identification’ with a social category increases ‘helping’ towards other members of that category. The strong previous evidence for a link between group identification and helping suggests that identification might function as a mediator, so that shared common fate increases identification, which in turn increases prosocial intentions towards ingroup members.

It was expected that these mechanisms would also hold true in the context of the COVID‐19 crisis. One feature that makes the pandemic interesting in theoretical terms is that it constitutes a rarely precedented situation, where the problems caused by the COVID‐19 pandemic threat are shared not only with co‐nationals within one's country, but with all humans globally. COVID‐19 poses a threat to humanity that does not stop at national borders. Because the COVID‐19 pandemic adversely impacts virtually all humans, albeit in unequal measure (see, e.g., BBC, 2020a), this devastating event allows us to test whether the perceived shared common fate on a global level increases identification with all humans (McFarland, Webb, & Brown, 2012), and prosocial tendencies towards all humans—including both national ingroup and outgroup members. ‘Identification with all of humanity’ is a construct that was introduced by McFarland et al. (2012) to capture an orientation whereby humanity is not perceived in terms of ethnic, religious or other divisions, but where it is perceived as one all‐inclusive group with all members being entitled to fair, non‐prejudiced treatment. Given that the pandemic has created a situation where humans can potentially develop an increased awareness of their mutual interdependence and globally shared threats, this situation is well suited to assessing the potency of the construct ‘all of humanity’ for shaping social behaviour.

Hence, it was expected that a perception that humanity faces a shared global common fate in the face of coronavirus would be related to greater identification with all of humanity, and that this variable would mediate the positive effects of perceived global common fate on all others suffering from the crisis, including both national ingroup members and national outgroup members, since regardless of nationality all humans are fellow ingroup members in the overarching category of humanity. A focus on global solidarity, in the form of global common fate and identification with all of humanity, was thus proposed to motivate prosocial acts both within but also across national group boundaries.

### 1.3 Factors related to national solidarity: National ‘closing ranks’

As indicated above, a survey of media coverage of political responses to the pandemic in Britain suggests that appeals by politicians to national solidarity have been much more frequent than appeals to global solidarity. Political leaders often use external threats as an opportunity to cement their own leadership by calling for the population to ‘rally round the flag’ and ‘close ranks’, that is, to move closer together, psychologically speaking, in the face of adversity (Reicher & Hopkins, 2001). Because external threats can be used by politicians to further their own agendas (Reicher & Hopkins, 2001), it is maybe no surprise that politicians in Britain have capitalized on this narrative, also in the context of COVID‐19. National identities are easily invoked (Billig, 1995), they are flagged by politicians and in the media through symbols and habits of language. In the context of the COVID‐19 pandemic, too, perceptions of threat are open to manipulation (Lalot, Abrams, & Travaglino, 2021), and they have important behavioural consequences, such as, indeed, increasing support for political leaders (Yam et al., 2020).

In Britain, such sentiments of national ‘closing ranks’, standing ‘shoulder to shoulder’ and demonstrating greater ‘national unity’ are often invoked by references to the ‘Dunkirk spirit’ – to this day, memories of Britain's war effort and success during WWII go a long way to engender feelings of national pride and unity in this country (e.g., The Evening Express, 2021; The Telegraph, 2021). The phrase, first used sometime in 1940, refers to the evacuation of the British troops from northern France after German military advances. The evacuation took place under constant air attack and using warships but also a flotilla of small civilian pleasure boats and civilian working barges. The successful evacuation was then referenced by politicians to raise British morale and this gave rise to the notion of ‘Dunkirk spirit’. The phrase implies refusal to surrender when Britain is faced with an external threat. It invokes nationalist sentiments and is hence an image popular with the political right, but it has also connotations of solidarity and ‘pulling together’ in times of adversity. As seen above, closer affinity and identification with a social group positively affects willingness to act prosocially towards others in that group. Because of this, appeals to the idea of ‘closing ranks’ at the national level in the face of the COVID‐19 threat can be assumed to be related to greater prosocial tendencies towards fellow national ingroup members. Greater solidarity at the national level implies greater psychological closeness with fellow nationals. Because prosocial behaviour is encouraged by greater perceived closeness to, similarity with, and oneness with the target of help, national ‘closing ranks’ should encourage help for national ingroup members suffering due to COVID‐19. After all, the idea that the national ingroup needs to ‘pull together’ to defeat an external threat does not only imply that oneself will be helped by other group members, but it also implies that oneself has an obligation to help other ingroup members.

At the same time, an appeal to national solidarity throws into sharp relief group boundaries that exclude those who are not part of the national ingroup. National solidarity clearly stipulates that certain actions and sacrifices are required for the benefit of the national ingroup, whereas national outgroups are defined as being outside of the moral sphere of obligation. Because of this, it is hypothesized that national ‘closing ranks’ would be positively related to help extended to fellow nationals, but that it would be *negatively* related to help extended to national outgroup members.

### 1.4 Summary of hypotheses, and the present research

In sum, then, it was proposed that a perception of global common fate in the context of the COVID‐19 pandemic would be positively associated with prosocial helping, in the form of donation intentions to benefit both national ingroup and outgroup members (Hypothesis 1). In contrast, it was proposed that a desire to ‘close ranks’ at the national level would be positively associated with donation intentions to benefit national ingroup members, but that it would decrease donation tendencies towards national outgroup members (Hypothesis 2). Moreover, it was hypothesized that the effect of global common fate on donations would be mediated by identification with all of humanity, so that shared fate would be positively associated with identification with all humans, which would in turn be positively associated with prosociality towards all humans, including both national ingroup and outgroup members (Hypothesis 3).

To test our predictions, two studies were conducted. The first study took place in October 2020, and the second study was conducted in November 2020, to replicate and confirm the effects. Predictions were identical across both studies, but two studies were conducted to meet standards for the demonstration of replicability of results. Study 1 asked British participants about their willingness to help fellow British nationals (ingroup members), and their willingness to help those suffering from the effects of the pandemic in China and Syria. China was chosen as an outgroup target of interest because the virus first emerged from China, making it an interesting comparison point. Syria was chosen because at the time of the study there were some actual charitable donation appeals by humanitarian charities focussing on the devastating impact of the pandemic on refugee populations.

## 2 STUDY 1

### 2.1 Method

#### 2.1.1 Participants

The sample consisted of 250 people recruited via the Prolific platform in October 2020 (mean age = 36 years, age range 18–70, 172 females, 77 males, 1 non‐binary person). All participants were required to have British nationality. The sample size was determined by the fact that effects stabilize around *N* = 250 (Schönbrodt & Perugini, 2013), and that this sample size generated sufficient power to similar effects in previous work on intergroup helping in the context of COVID‐19 (James & Zagefka, 2017; Zagefka, 2021). Moreover, an a priori power analysis with G*Power (Faul, Erdfelder, Lang, & Buchner, 2007) was conducted, assuming a slope of .16 and *α* = .05 and aiming for a power of 0.80. This recommended *N* = 237, and we conservatively recruited *N* = 250 to be sure to achieve at least this desired level of power.

#### 2.1.2 Measures

All measures were assessed with Likert scales (1 = ‘strongly disagree’ to 5 = ‘strongly agree’). To start with, participants were told that the survey questions all related to the COVID‐19 crisis.

Perceived *global common fate* was measured with four items: ‘We need to address coronavirus as a global community’, ‘Citizens of the world need to address the coronavirus problem together’, ‘The British success in addressing coronavirus depends on global cooperation’, and ‘The British fate is interlinked with that of other countries’, *α* = .77.

*National ‘closing ranks’* was measured with three items: ‘When threatened from outside, the British people must stick together’, ‘In the face of an external threat, we British must rally round the flag’, and ‘When facing threats from outside Britain, we must all do our bit to defend the British people’, *α* = .83.

*Identification with all of humanity* was measured with two items inspired by McFarland et al.'s scale (2012): ‘I identify with all humans’, and ‘I see myself as a citizen of the world’, *α* = .65.

*Donations to ingroup victims* was measured with two items previously used by Zagefka, Noor, Randsley de Moura, Hopthrow, and Brown (2011): ‘I would be willing to donate money to British coronavirus victims’, and ‘I think it is important to donate money to British coronavirus victims’, *α* = .82.

*Donations to outgroup victims* were measured with items worded like the ones above, but this time asking about donations to Chinese coronavirus victims and coronavirus victims in Syria, *α* = .92 for this 4‐item measure. The order in which ingroup and outgroup donations were measured was randomized.^1^

All aspects of this and the subsequent studies were in line with British Psychological Society ethics guidelines. The data for this study and Study 2 can be found here: https://osf.io/m67fa/?view_only=a66ce2de2d3c49c39399bcecdf1f1959.

### 2.2 Results and discussion

Bivariate correlations and means are displayed in Table 1 (values below the diagonal). To test whether perceived global common fate and national ‘closing ranks’ would have the predicted effects on ingroup and outgroup helping, that is, H1 and H2, respectively, two linear regressions were run with these two variables as predictor variables. Results are displayed in Table 2.

**TABLE 1:** Bivariate correlations and means, Studies 1 (*N* = 250) and 2 (*N* = 250)

	1. Donations to ingroup victims	2. Donations to outgroup victims	3. National ‘closing ranks’	4. Global common fate	5. Identification with all of humanity
1. Donations to ingroup victims		.55***	.18**	.21***	.20***
2. Donations to outgroup victims	.52***		−.17**	.15*	.20***
3. National closing ranks	.12*	−.33***		.09	.06
4. Global common fate	.10	.14*	−.001		.31***
5. Identification with all of humanity	.16**	.30***	−.18**	.52***	
Study 1 Means	3.20	2.81	3.61	4.30	4.08
Study 1 *SD*	0.89	0.99	0.86	0.62	0.78
Study 2 Means	3.17	2.64	3.38	3.89	3.97
Study 2 *SD*	0.99	1.00	0.89	0.74	0.76

*Note*: Study 1 correlations below the diagonal and Study 2 correlations above the diagonal. The scale for both studies ranged 1–5 with 5 indicating strong endorsement of the construct.

* *p* < .05;

** *p* < .01;

*** *p* < .001.

**TABLE 2:** Regression results predicting ingroup and outgroup helping from perceived global common fate and national closing ranks, Studies 1 (*N* = 250) and 2 (*N* = 250)

	DV: Donations to ingroup victims				DV: Donations to outgroup victims			
	Standardized beta	Unstandardized *B*	Lower confidence interval	Upper confidence interval	Standardized beta	Unstandardized *B*	Lower confidence interval	Upper confidence interval
Study 1 results								
Perceived global common fate	.10	.15	−.04	.33	.14*	.23	.05	.43
National ‘closing ranks’	.12*	.13	.01	.26	−.33***	−.38	−.55	−.24
Study 2 results								
Perceived global common fate	.20**	.26	.08	.44	.16*	.22	.04	.42
National ‘closing ranks’	.16*	.18	0.20	0.34	−.19**	−.21	−.36	−.07

* *p* < .05;

** *p* < .01;

*** *p* < .001.

When predicting ingroup donations, the overall model was significant, *F*(2,247) = 3.27, *p* < .05, *R* ^2^ = 0.03, and this was also the case when predicting outgroup donations, *F*(2,247) = 18.18, *p* < .001, *R* ^2^ = 0.13. As is apparent, in line with H1 perceived global common fate was positively related to donations. However, although according to H1 positive associations were expected for both outgroup and ingroup helping, the association with ingroup helping did not reach significance. In line with H2, national ‘closing ranks’ was positively related to ingroup donations, but *negatively* related to outgroup donations.

**FIGURE 1:**
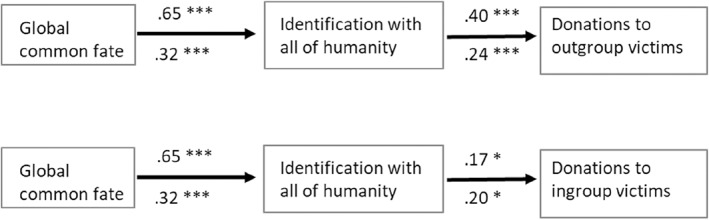
Identification with all of humanity mediates the effect of perceived global common fate on ingroup and outgroup helping. Unstandardized path coefficients for Study 1 (*N* = 250) above the arrows and for Study 2 (*N* = 250) below the arrows. ***p < .001

To test H3, whether the effect of ‘perceived global common fate’ on ‘outgroup donations’ was mediated by ‘identification with all of humanity’, Hayes' (2018) PROCESS macro Model 4 was used. Perceived global common fate was related to identification with all of humanity, *b* = 0.65, *SE* = 0.07, 95% CI [0.67; 1.85]. Identification with all of humanity was, in turn, related to outgroup donations, *b* = 0.40, *SE* = 0.09, 95% CI [0.22; 0.57]. As expected, there was an indirect effect of global common fate on outgroup donations, *b* = 0.26, *SE* = 0.06, 95% CI [0.14; 0.38]. Results are illustrated in Figure 1.^2^

To test the second part of H3, whether the effect of ‘perceived global common fate’ on ‘ingroup donations’ was mediated by ‘identification with all of humanity’, again Hayes' (2018) PROCESS macro Model 4 was used. Perceived global common fate was related to identification with all of humanity, *b* = 0.65, *SE* = 0.07, 95% CI [0.52; 0.79]. Identification with all of humanity was, in turn, related to ingroup donations, *b* = 0.17, *SE* = 0.08, 95% CI [0.007; 0.33]. As expected, there was an indirect effect of global common fate on ingroup donations, *b* = 0.11, *SE* = 0.06, 95% CI [0.004; 0.24].^3^

Taken together, clear evidence was found for all three hypotheses. Perceived global common fate was positively associated with help for outgroup members and ingroup members, although the latter did not reach significance (H1). In contrast, a desire to ‘close ranks’ at the national level was positively associated with help for national ingroup members but it did also have a strongly negative relationship with help for outgroup members (H2). In other words, while an emphasis on global solidarity had positive associations with prosociality within and across group boundaries, national solidarity had positive associations with prosociality within the ingroup but it was negatively related to prosociality with outgroup members. This suggests that in order to foster maximum benefits in terms of prosociality, one would be well advised to favour a global perspective over a national perspective.

Mediation analyses confirmed that the effect of perceived global common fate on help for outgroup members and ingroup members was indeed due to a greater degree of identification with all of humanity (H3). As expected, a perception of having a common destiny and fortune was positively associated with participants feeling closer to and more connected with national outgroup members, which in turn was positively linked to prosocial tendencies towards them. These results support the idea that having a common destiny or facing a common adversity can help redefine identities in such a way that former outgroup members become included in a common ingroup, and therefore become part of one's moral ingroup and deserving of altruistic assistance. Following these encouraging results, a second study was run to confirm the robustness of the effects.

## 3 STUDY 2

### 3.1 Method

#### 3.1.1 Participants

A second sample of 250 people was recruited via the Prolific platform in late November 2020 (mean age = 35 years, age range 18–77, 169 females, 80 males, 1 differently identified). All participants were required to have British nationality. The sample size was designed to match that of Study 1.

#### 3.1.2 Measures

All measures were assessed with Likert scales (1 = ‘strongly disagree’ to 5 = ‘strongly agree’). To start with, participants were told that the survey questions all related to the COVID‐19 crisis.

Perceived *global common fate*, *national ‘closing ranks’*, and *identification with all of humanity* were measured with items very similar or identical to those used in study 1, *α* = .78 for the four item measure of *global common fate*, *α* = .83 for the three item measure of *national ‘closing ranks’*, and *α* = .69 for the two item measure of *identification with all of humanity*.

*Donations to ingroup victims* was measured with three items, preceded by the question ‘Would you be willing to donate money to help people suffering from the coronavirus crisis?’: ‘I would donate to help British people in trouble’, ‘I would want to offer financial support to British people in trouble’, and ‘Donating money to British people is the right thing to do’, *α* = .95.

*Donations to outgroup victims* were measured with the same three items, but this time tailored to the target of Chinese people suffering due to the COVID‐19 crisis, *α* = .95. The order in which ingroup and outgroup donations were measured was randomized. Because the two outgroups in Study 1 yielded very similar responses, to the point that it was sensible to not treat them separately, in Study 2 a decision was made to only focus on one outgroup.

### 3.2 Results and discussion

Bivariate correlations and means are displayed in Table 1 (values above the diagonal). To test whether perceived global common fate and national ‘closing ranks’ would have the predicted effects on ingroup and outgroup helping, respectively, again two linear regressions were run with these two variables as predictor variables. Results are displayed in Table 2.

When predicting ingroup donations, the overall model was significant, *F*(2,247) = 9.26, *p* < .05, *R* ^2^ = 0.07, and this was also the case when predicting outgroup donations, *F*(2,247) = 7.35, *p* < .001, *R* ^2^ = 0.06. Again, perceived global common fate was positively related to outgroup donations and ingroup donations (significantly so for both target groups), whereas national ‘closing ranks’ was positively related to ingroup donations, but *negatively* related to outgroup donations. Taken together, again H1 and H2 were supported.

To test whether the effect of perceived global common fate on ‘outgroup donations’ was mediated by identification with all of humanity (H3), again Hayes' (2018) PROCESS macro Model 4 was used. Mirroring the results of the previous study, perceived global common fate was again related to identification with all of humanity, *b* = 0.32, *SE* = 0.06, 95% CI [0.20; 0.44]. Identification with all of humanity was, in turn, related to outgroup donations, *b* = 0.24, *SE* = 0.09, 95% CI [0.07; 0.41]. As expected, again there was an indirect effect of global common fate on outgroup donations, *b* = 0.08, *SE* = 0.03, 95% CI [0.02; 0.15]. Results are illustrated in Figure 1.

Testing, with Hayes' Model 4, whether the effect of perceived global common fate on ‘ingroup donations’ was mediated by identification with all of humanity (H3), perceived global common fate was related to identification with all of humanity, *b* = 0.32, *SE* = 0.06, 95% CI [0.20; 0.44]. Identification with all of humanity was, in turn, related to ingroup donations, *b* = 0.20, *SE* = 0.08, 95% CI [0.03; 0.36]. As expected, there was an indirect effect of global common fate on ingroup donations, *b* = 0.06, *SE* = 0.03, 95% CI [0.01; 0.13].^4^

Taken together, again there was clear support for all three hypotheses. Perceived global common fate was positively associated with prosociality towards both ingroup and outgroup targets (H1), whereas national ‘closing ranks’ was positively associated only with prosociality towards ingroup targets, but it was *negatively* associated with prosociality with outgroup targets (H2). Once more, the effects of global common fate on both ingroup and outgroup helping were mediated by identification with all of humanity (H3).

## 4 OVERALL DISCUSSION

Results are in line with the notion that a perception of global common fate increases donation intentions that benefit both national ingroup and national outgroup members and that it does so via enhanced identification with all of humanity, whereas a desire to ‘close ranks’ at the national level increases donations intentions that benefit national ingroup members but *decreases* helping extended across national group boundaries. These findings have some important theoretical but also practical implications.

In terms of theoretical implications, the findings confirm the usefulness of the category ‘all of humanity’. This construct has previously been introduced and measured to explain a range of intergroup phenomena, for example, the actions of those who helped Jews during the Nazi regime in Europe (McFarland, Brown, & Webb, 2013). However, there has rarely been a situation where all of humanity is facing the same immediate threat as is the case with the current pandemic. This article capitalized on the opportunity to demonstrate that identification with all of humanity can be triggered by perceived global common fate, and that it might be particularly important for psychological responses to events that can be construed as a threat at either the national or global level. Another example of such a threat could be climate change.

A further theoretical implication is that the findings confirm the importance of identity processes in informing people's helping decisions. The findings are in line with what would be expected on the grounds of social identity theory (Tajfel & Turner, 1986): if the ingroup is perceived as facing a threat, this often increases identification with the group and also prosociality towards ingroup members. COVID‐19 is undoubtedly one such threat that can be expected to generate such processes (Jetten et al., 2020).

Relatedly, another theoretical implication is that the same theoretical predictors of prosociality can have confluent or divergent effects on ingroup and outgroup helping. In addition to once again underscoring the importance of social identities for the study of prosociality, this means that group memberships might not only have direct, main effects on the extent of help, but that they might also *moderate* the effects of variables associated with prosociality (on the idea of group memberships as moderators, see also Stürmer et al., 2005). In other words, the findings suggest that considering group memberships in the context of prosociality can lead to a more nuanced picture than approaches that focus merely on individual differences and intrapersonal processes.

Last but not least, the present work goes beyond the existing literature in one important way. It tests, simultaneously, the concurrent roles of (a) threat external to the ingroup and (b) threat external to both the ingroup and the outgroup, as predictors of ingroup and outgroup helping. This dual focus is novel, and the findings present some initial insights which speak to the implications for prosociality when considered within a framework that incorporates both threat and multiple levels of categorization.

Having said this, maybe even more important than the theoretical implications are practical implications of these findings. These are twofold: insights for the generation of charitable donations more generally, and insights for behaviour change in the context of the COVID‐19 pandemic.

In general terms, charitable organizations need to understand what motivates people to donate to charitable causes. Psychological factors that drive donations can be used to design more effective fundraising appeals. The present findings confirm that highlighting commonalities between potential donors and recipients of donations will be beneficial. Any message that highlights camaraderie and unity between the two entities should positively affect donation proclivity. Appeals to solidarity, and the idea of unity in the face of common threat and fate, are likely to increase donations. Moreover, of course different fundraising appeals are often in stiff competition with one another, and the findings highlight that measures that elicit donations at one level of categorization (e.g., the national ingroup) will often have detrimental effects at other levels of categorization (e.g., national outgroups). Careful consideration of funding priorities that takes into account these interdependent effects is therefore advisable.

Having said this, maybe the most important practical implications of the present findings are specific to the COVID‐19 situation. In agreement with Drury, Carter, Ntontis, and Guven (2021), the findings confirm that group processes can be harnessed to bring about behaviour change in the context of this pandemic, and this present work applied this general principle to the realm of prosociality and donations designed to mitigate the impacts of the pandemic. Appeals that emphasize certain unique features of the COVID‐19 situation, for example, goal interdependence between different countries and the fact that all humans are united in facing the same adversity, are likely to increase willingness to donate to the COVID‐19 response both at home *and* abroad.

Clearly, there is a mismatch between the prevailing political rhetoric regarding the pandemic, and the messaging that can be expected to bring the greatest benefits in the context of this global pandemic. British politicians are emphasizing national unity, which was shown here to have detrimental effects on willingness to extend help across national boundaries. Yet, as seen above, the only feasible way out of the pandemic is international collaboration. Solidarity with those suffering the impacts of the coronavirus in the world's poorest countries is not only vital because of principles of fairness and human decency, but it is also in the self‐interest of the rich nations: as repeatedly emphasized by health experts, vaccinating an entire wealthy nation is not going to bring long‐term solutions when the virus is allowed to spread freely in other parts of the world, mutilate there, and boomerang back (The Guardian, 2020). In order to foster support for the idea of global cooperation and mutual international help, messages that focus on global solidarity are needed, rather than the messages that focus on national solidarity. The implications of the present results are that in order to generate support for measures that are needed to overcome the crisis, the political rhetoric needs to move away from a focus on national unity, and put more emphasis on the need for global solidarity. National protectionism is not going to be a viable solution for managing this global pandemic, which requires a global solution.

The present work has, of course, some important limitations, which at the same time point to avenues for further research and improvement. Most importantly, the data are correlational, which means that they cannot support any claims about the causal direction of observed effects. Further, experimental data would be needed for that. Moreover, the present research only studied self‐reported intentions to donate, rather than actual donation behaviour. Therefore, a degree of uncertainty remains about whether the processes identified here would also affect donation behaviour. Having said this, good evidence exists that often self‐reports are a valid proxy for actual behaviour. Although people tend to over‐report helping compared to actual behaviour in terms of mean levels, self‐reports and actual behaviour are quite highly correlated (Bekkers & Wiepking, 2011; Zagefka et al., 2011). Therefore, self‐reports are likely to be a good proxy for actual behaviour in studies like the present which assess correlational patterns. Nonetheless, future work could expand on the present design by incorporating a measure of actual behaviour.

In addition to addressing the methodological limitations acknowledged above, future work could also address some interesting conceptual issues. Most importantly, it is not clear from this study whether the processes found here are specific to COVID‐19, or whether they would emerge for other challenges that can be perceived as being shared by national ingroup members or indeed all of humanity. Other such challenges exist, with climate change being a prime example. Future work would be needed to establish in how far the processes generalize to other global problems.

In sum, the findings suggest that a focus on global solidarity is preferable over national solidarity, not only on the ground of humanitarian principles, but also because only a global focus is well aligned with an international solution to the crisis, which is the only feasible solution according to health experts. Given that the pandemic is, at present, causing simultaneously an increased need for funds to address COVID‐19‐related problems and a reduction in available funds due to a global economic downturn, it is hoped that the psychological mechanisms described here can be useful to increase the effectiveness of fundraising appeal design, and through this the management of the global pandemic.

## Supporting information


**Data S1.** Supporting information.

## Data Availability

The data for the study can be found here: https://osf.io/m67fa/?view_only=a66ce2de2d3c49c39399bcecdf1f1959

## Endnotes

^1^
The questionnaires for both studies also included questions such as about blame attributions for the crisis and anxiety related to the crisis. The study was designed to test both the processes outlined here and, separately, a hypothesis about blame attributions and how they relate to helping. Blame attributions are not relevant for the present hypotheses and will not be discussed further here.

^2^
In theory, it would be possible to test both hypotheses jointly using Structural Equation Modelling (SEM). However, ingroup donations and outgroup donations tend to be very highly correlated, as indeed they should be, given that both are strongly driven by dispositional tendencies for altruism and empathy (see, e.g., Politi et al., 2021). Therefore, including them simultaneously as unrelated outcome variables in one model is not feasible. Although it would be possible to estimate two SEMs, one for each outcome variable, this would add nothing over and above the regression and mediation analyses presented here.

^3^
Two structural equation models (SEMs) were also estimated, one predicting ingroup help and the other outgroup help. Given that across the three hypotheses there are two main predictors and one mediator, the goal was to consider both exogenous variables simultaneously when testing for mediation, to avoid risking inflation of the mediation hypothesis. In the SEMs, ‘global common fate’ and ‘national closing ranks’ were the exogenous variables which were allowed to covary. The model specified an indirect effect of global common fate on helping via ‘identification with all of humanity’, and it also allowed for both an indirect effect (via identification with all of humanity) as well as a direct effect of ‘national closing ranks’ on helping. Both models fitted the data well (*χ* ^2^s ≤ 5.57, CFIs ≥ 0.90, RMSEAs ≤ 0.001). Crucially, the indirect effects of ‘global common fate’ were significant in both instances, with the standardized effect being 0.10, *p* < .02 when predicting ingroup helping, and 0.13, *p* < .02 when predicting outgroup helping.

^4^
Again, SEMs tested, as for the previous study, if the indirect effects of global common fate on helping would still be significant in a model that also simultaneously included ‘national closing ranks’. Again, this was the case, with the standardized indirect effect being 0.06, *p* < .004 when predicting ingroup helping, and 0.07, *p* < .008 when predicting outgroup helping.
