# Supplementary material for: Prosociality during COVID‐19: Globally focussed solidarity brings greater benefits than nationally focussed solidarity
Source: J Community Appl Soc Psychol. 2021 Jun 16;32(1):73–86. doi: 10.1002/casp.2553 (PMC8426876; doi:10.1002/casp.2553)
Supplement: Supplementary file 1 — Data S1. Supporting information. [file CASP-32-73-s001.pdf]

Prosociality during COVID-19: Globally focussed solidarity brings greater benefits than nationally focussed solidarity

Prof. Hanna Zagefka

Community and Social Impact Statement

Relevance of research to communities

- Two correlational studies investigated what drives donations and solidarity with people suffering due to the Covid-19 pandemic
- The research tested drivers of help offered to members of one's immediate community (the national ingroup) and members of other communities (national outgroups)
- Key findings:
  - Those who are more conscious of global common fate and the interdependence of all of humanity in the face of the virus are more likely to identify with others beyond national group boundaries, and they are more likely to show solidarity with others irrespective of national identities
  - In contrast, rhetoric focussed on intra-national solidarity (as often invoked by national politicians) was positively associated with solidarity with national ingroup members, but it was *negatively* associated with solidarity beyond national group boundaries.

Practical implications for community settings

- Community leaders and politicians should be mindful that messages designed to encourage solidarity within communities can unintentionally reduce solidarity with those outside of community boundaries
- In the context of Covid-19, messaging that focussed on *global* solidarity is likely to have more beneficial effects than messaging that focusses on intra-community solidarity only.
